# Heterogeneity and plasticity of epithelial–mesenchymal transition (EMT) in cancer metastasis: Focusing on partial EMT and regulatory mechanisms

**DOI:** 10.1111/cpr.13423

**Published:** 2023-02-19

**Authors:** Dandan Li, Lingyun Xia, Pan Huang, Zidi Wang, Qiwei Guo, Congcong Huang, Weidong Leng, Shanshan Qin

**Affiliations:** ^1^ Department of Stomatology, Taihe Hospital and Hubei Key Laboratory of Embryonic Stem Cell Research, School of Basic Medical Sciences Hubei University of Medicine Shiyan China; ^2^ Laboratory of Tumor Biology, Academy of Bio‐medicine Research Hubei University of Medicine Shiyan China

## Abstract

Epithelial–mesenchymal transition (EMT) or mesenchymal–epithelial transition (MET) plays critical roles in cancer metastasis. Recent studies, especially those based on single‐cell sequencing, have revealed that EMT is not a binary process, but a heterogeneous and dynamic disposition with intermediary or partial EMT states. Multiple double‐negative feedback loops involved by EMT‐related transcription factors (EMT‐TFs) have been identified. These feedback loops between EMT drivers and MET drivers finely regulate the EMT transition state of the cell. In this review, the general characteristics, biomarkers and molecular mechanisms of different EMT transition states were summarized. We additionally discussed the direct and indirect roles of EMT transition state in tumour metastasis. More importantly, this article provides direct evidence that the heterogeneity of EMT is closely related to the poor prognosis in gastric cancer. Notably, a seesaw model was proposed to explain how tumour cells regulate themselves to remain in specific EMT transition states, including epithelial state, hybrid/intermediate state and mesenchymal state. Additionally, this article also provides a review of the current status, limitations and future perspectives of EMT signalling in clinical applications.

## Graphical abstract

- The general biomarkers in EMT signalling pathway were updated.
- Cellular EMT transition state is continuous, including epithelial state, diverse hybrid states (p‐EMT states) and mesenchymal state. Tumour cells in p‐EMT state may play a role in hematogenous metastasis due to their extreme plasticity.
- We propose a seesaw model between EMT drivers and MET drivers to explain how cells regulate their own EMT transformation state. The expression of EMT drivers and MET drivers is finely regulated by the double negative feedback loop in EMT signalling.
- Tumour heterogeneity should be partly attributed to the EMT plasticity of tumour cells. The inter‐tumour heterogeneity is clinical associated with poor prognosis in gastric cancer.
- EMT signalling plays crucial roles in cancer metastasis, stemness, chemoresistance and immune suppression. Targeting EMT signalling is a promising anti‐tumour strategy that kills numerous birds with one stone.

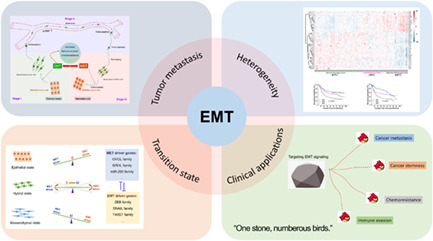

## 1 INTRODUCTION

**FIGURE 1:**
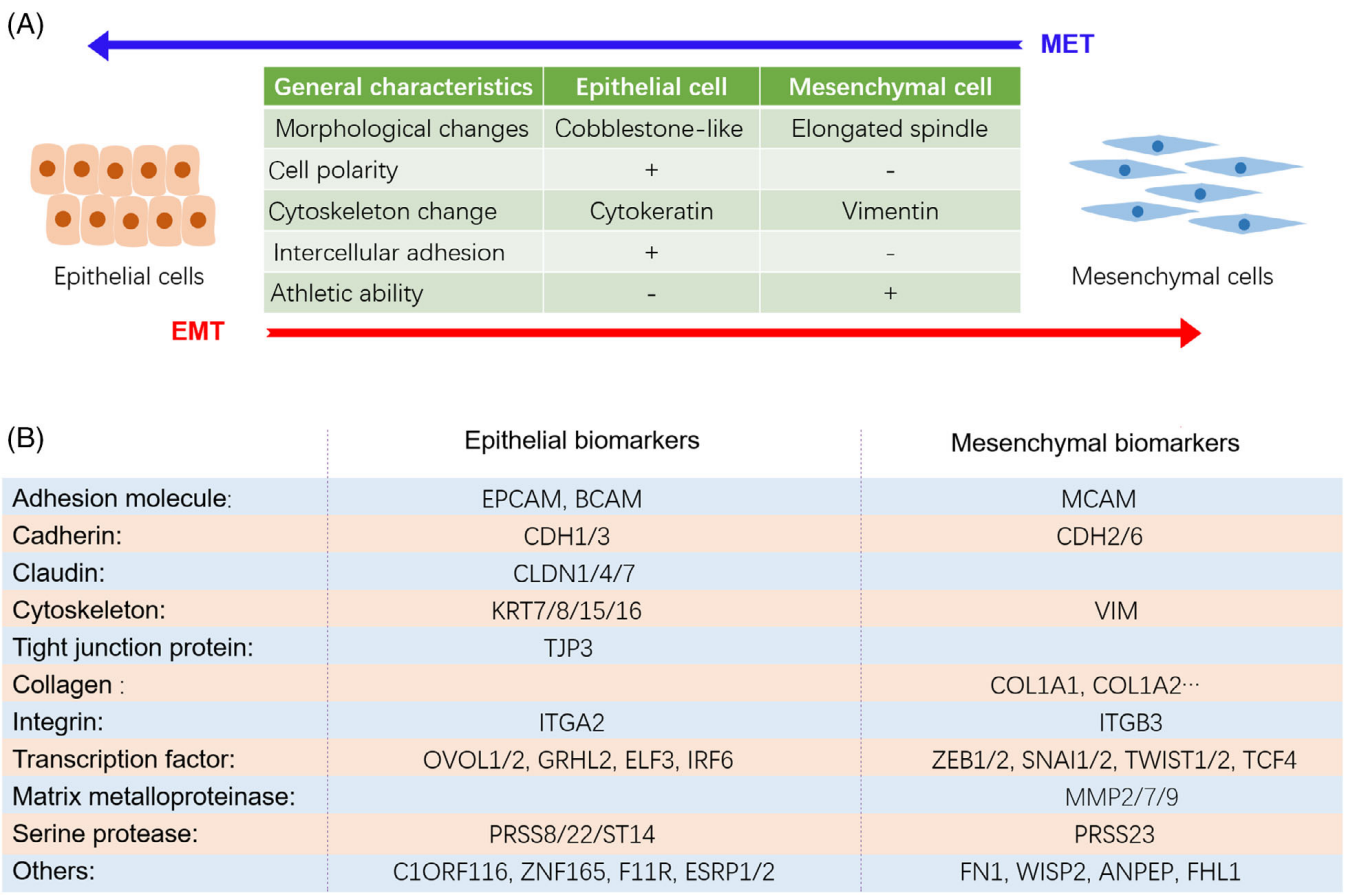
The general characteristics and biomarkers of cellular EMT process. (A) Typical features of cells undergoing EMT. (B) EMT biomarkers were further updated and classified by analyzing the transcriptome data of cells before and after EMT.

Epithelial–mesenchymal transition (EMT) is essentially a biological process in which cells switch from epithelial phenotype to mesenchymal phenotype,^1^ accompanied by multiple characteristic changes (Figure 1A), including but not limited to morphological alterations, loss of cell polarity, cytoskeleton changes and intercellular de‐adhesion as well as acquiring the ability to invade and exercise.^2^ The reduction of intercellular adhesion and the enhancement of migration characteristics induced by EMT plays critical roles in promoting tumour cells invasion and migration.^3^, ^4^, ^5^ For examples, Sharaireh et al. reported that loss of E‐cadherin (E‐cad) junctions is an early event of EMT during cellular transformation.^6^ Vimentin, a protein associated with cell motility, is recognized as the heart of EMT‐mediated metastasis.^7^ It is reported that long non‐coding RNA VAL promotes EMT‐independent metastasis through by regulating the protein stability of Vimentin.^8^

As a naturally occurring transdifferentiating program, cellular EMT events can be defined by EMT‐related biomarker.^9^ These biomarkers can be roughly divided into two categories, epithelial markers and mesenchymal markers. The occurrence of EMT is commonly accompanied by a significant down‐regulation of epithelial markers and a significant up‐regulation of mesenchymal markers.^9^, ^10^, ^11^ Herein, we updated EMT‐related biomarkers by analysis of the transcriptome data (GSE81167, GSE70551, GSE43489 and GSE214471) in HCC827, MCF10A, PC3, SGC7901 and AGS cell lines, respectively.^12^, ^13^, ^14^ According to the bulk RNA‐seq analysis, in addition to well‐known adhesion molecules (EPCAM and BCAM), cadherins (CDH1/2/3/6), claudins (CLDN1/4/7), MMPs (MMP2/7/9), epithelial splicing regulatory proteins (ESRP1/2), EMT‐related transcription factors (ZEBs, Twists, Snails, OVOLs and GRHLs), we also found a group of serine proteases (PRSS8/22 and ST14) whose expressions were significantly altered during EMT.

Several independent research teams have successively confirmed that the ZEB1‐driven EMT process in lung cancer cells was accompanied by a decrease in the expression of the epithelial cell adhesion molecule E‐cadherin and EPCAM and an increase in the expression of the cytoskeletal protein vimentin.^14^, ^15^, ^16^ Analysis of the EMT‐related transcriptome data showed that multiple epithelial serine protease genes, including PRSS8, PRSS22 and ST14, were dramatically altered in EMT/MET progression, suggested these epithelial serine proteases PRSS8, PRSS22 and ST14 can serve as biomarkers of EMT signalling. Among those EMT‐related serine proteases, PRSS8 has been reported to inhibit EMT signalling in colon, bladder and lung cancer.^17^, ^18^, ^19^, ^20^ ST14, encoding a type II transmembrane serine protease matriptase, was reported to be a ZEB1‐responsive gene in lung cancer.^15^ PRSS22 was an epithelium serine protease tryptase ϵ that expressed in airway epithelial cells.^21^, ^22^ Conversely, PRSS23 was a mesenchymal serine protease extremely co‐expressed with mesenchymal markers and cancer‐associated fibroblasts markers in gastric cancer.^23^ In addition, studies have shown that epithelial biomarkers, such as S100A14, ZNF165 and C1ORF116, were heavily altered in expression during EMT/MET.^24^, ^25^, ^26^, ^27^ Based on EMT‐related transcriptome analysis and recent advances, we herein have updated the EMT‐related biomarkers in Figure 1B.

Notably, EMT is not a binary process, but contains various intermediate states (also known as hybrid state) or partial EMT (p‐EMT) states.^28^, ^29^, ^30^ Recently, single cell RNA sequencing has confirmed the continuous EMT transition states including p‐EMT in an individual tumour.^31^ Simeonov et al. found that the tumour population has achieved full coverage from epithelial state to complex and diverse hybrid states (p‐EMT state) and then to mesenchymal state.^31^ Notably, the cells in the hybrid states possessed strong characteristics of both pro‐invasion and pro‐tumour, and account for the vast majority of tumour cells.^32^ Compared with epithelial and mesenchymal cells, p‐EMT state cells with high plasticity may contribute more to tumour progression.^33^ This review focuses on the recent advances regarding EMT heterogeneity and plasticity in cancer metastasis, especially the hybrid epithelial/mesenchymal (E/M) state.

## 2 EMT AND TUMOUR METASTASIS

The clinical manifestations of malignant tumours vary depending on the organ, location and degree of development.^34^, ^35^ However, most malignant tumours have no obvious clinical symptoms in the early stage, and are often diagnosed at advanced stage, leading to poor survival.^36^ Metastasis is known to be the leading cause of cancer morbidity and mortality.^37^ Metastasis involves the spread of tumour cells from the primary site to metastatic focus, including surrounding tissues and distant organs.^36^ Since tumour metastasis is often characterized by multiple and diffuse distribution, the vast majority of patients have lost the opportunity of surgical treatment by this time.^38^

The tumour metastastic cascade is a complex biological process, which can be roughly divided into three stages, including (i) local invasion stage, (ii) hematogenous spread stage and (iii) distant colonization stage.^39^, ^40^, ^41^ In the local invasion stage, tumour cells first detach from the primary tumour site and then invade adjacent tissues and penetrate basement membrane. In the hematogenous spread stage, tumour cells enter the circulatory and lymphatic systems, survive under anoikic conditions and evade immune attack to become circulating tumour cells. In the distant colonization stage, tumour cells extravasate at distant capillary beds to form micrometastatic nodules, and then reprogram the surrounding stroma, and form macrometastases.^42^

**FIGURE 2:**
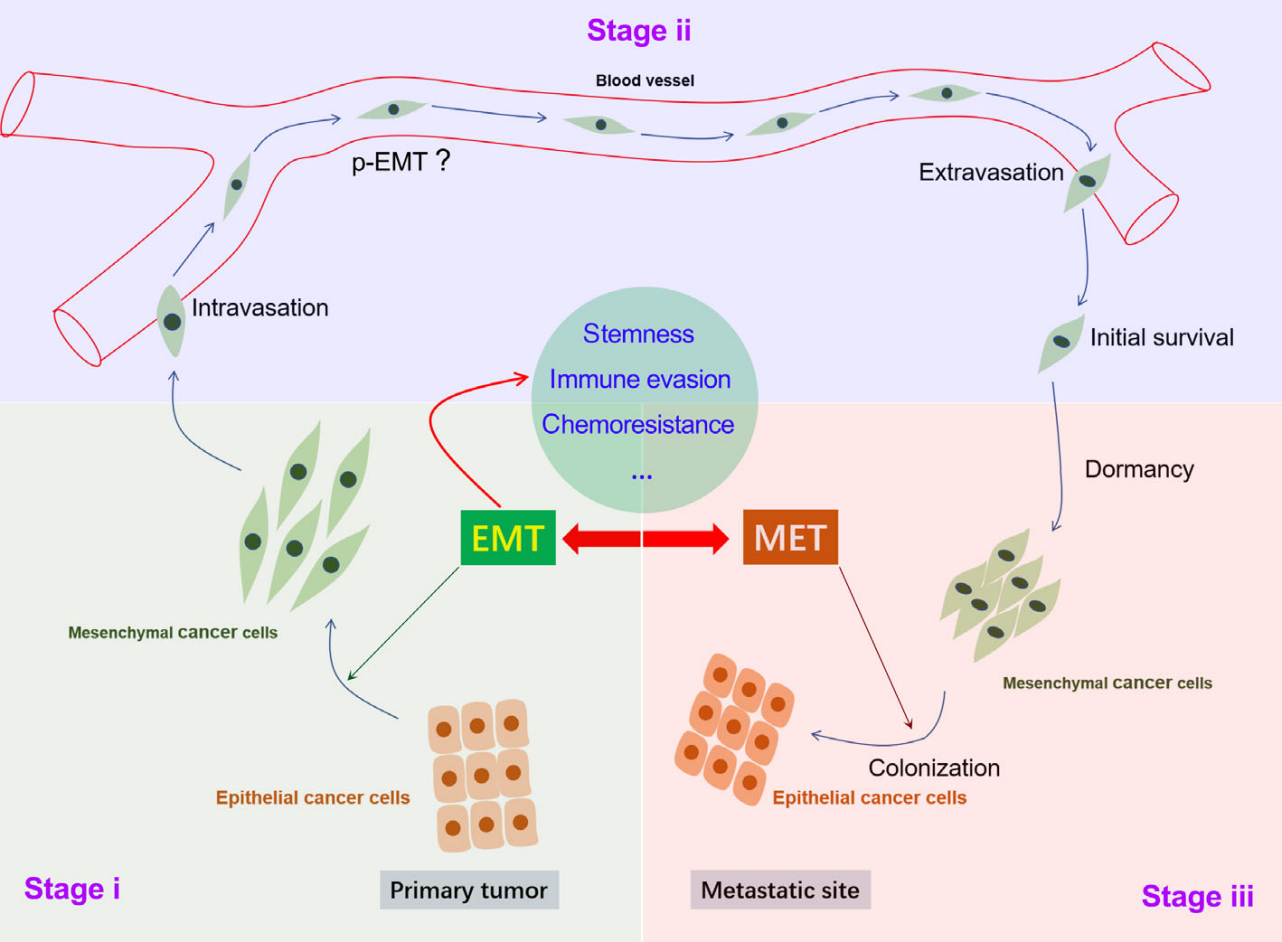
The role of EMT and MET in tumour metastasis.

More than 90% of human malignant solid tumours originate from epithelia.^43^ EMT signalling is known to play a critical role in cancer metastasis.^44^, ^45^, ^46^ It is generally believed that EMT process is required at the local invasion stage, while the MET program is required at the distant colonization stage (Figure 2). During the initial steps of the metastatic cascade, EMT is considered to be an essential step by which epithelial‐derived malignant tumour cells acquire the ability to migrate and invade.^47^ The occurrence of EMT helps cancer cells shed from the primary site and invade adjacent tissues.^48^, ^49^, ^50^ Meanwhile, these migratory and invasive mesenchymal‐like cells usually also acquire cancer stem cell properties and therapy resistance, leading to poor prognosis in patients with cancer.^51^ In contrast, the mesenchymal–epithelial transition (MET) program facilitates the colonization of tumour cells in distant organs, so that distant metastases often exhibit the epithelial structural features of their corresponding tissue of origin.^52^ For example, the liver, lung and thymus metastases of pancreatic cancer usually present typical features of pancreatic glands and duct‐like structures.^53^

The p‐EMT program plays an essential role in helping tumour cells survive in the hematogenous spread stage. Once entering the circulation system, most of the migrated tumour cells will die due to anoikis or immune attack. Cell plasticity is closely related to immune escape, drug resistance and cancer stemness.^54^ As summarized by Pastushenko et al., compared with cells in epithelial or mesenchymal state, cancer cells in p‐EMT states possessed the strongest plasticity.^55^ To a substantial content, such a strong plasticity of tumour cells in p‐EMT state will be helpful to increase the chance of survival from anoikis or immune attack or mechanical shear stress in circulation system, thereby completing distant metastasis.

Since p‐EMT appears to have distinct advantages in migration, survival in the bloodstream and seeding and propagation within secondary metastatic sites,^56^ it is reasonable to conduct that p‐EMT may be involved in Paget's “seed and soil” theory during metastasis. Distant metastasis of tumour is known to be organ‐specific.^57^ For example, the most common route of GC metastasis is lymph node metastasis, followed by peritoneal dissemination metastasis and liver metastasis.^58^, ^59^ According to Paget's “seed and soil” theory of cancer metastasis, the growth of “seed” (tumour cells) requires appropriate “soil” (tumour microenvironment).^60^ The process of “seeds” reaching the “soil” requires the selection of circulatory and lymphatic systems, and only a few “seeds” can reach specific “soil.” The p‐EMT states are essentially a general term for complex and diverse heterogeneous states between epithelial and mesenchymal states. Cells exhibiting p‐EMT states have greater metastatic competence than those characterized by either epithelial state or mesenchymal state.^33^ It is possible that the p‐EMT state of cells that successfully gone through the stage of hematogenous spread may be distinct in different tumours. In other words, these p‐EMT tumour cells that have successfully gone through the stage of hematogenous spread (“seed”) may need to choose a distinct tumour microenvironment (“soil”) that is beneficial to their own colonization at specific distant organ.

The key role of p‐EMT in tumour metastasis has been well documented. Weinberg et al. have demonstrated that it is the individual cells residing in p‐EMT state that determine the tumorigenicity of breast cancer. In addition, several independent single‐cell studies have shown that p‐EMT state is closely associated with tumour metastasis. For example, Puram et al. have confirmed that p‐EMT is significantly associated with lymph node metastasis.^61^ Similarly, Zhang et al. have found that patients in the p‐EMT high group had significantly lower progression‐free survival and higher mortality than patients in the p‐EMT low group, especially those who did not receive radical resection or radiotherapy in skull base chordoma.^62^ Pastushenko et al. have shown that knockout of protocadherin Fat1 in mice accelerates tumour initiation and malignant progression by induction of a p‐MET state in skin squamous cell carcinoma and lung tumours.^63^

In addition to directly conferring tumour cells with the ability to metastasis, EMT can also drive tumour metastasis indirectly through enhancing the stemness, immune evasion and chemoresistance of cancer cells.^64^ Growing evidences support the role of EMT signalling in cancer stemness, chemoresistance and immune suppression.^65^, ^66^, ^67^, ^68^ For example, multiple studies have reported that EMT drivers, such as SNAI2, ZEB1 and Twist1, play critical roles in cancer cell stemness in cancers.^69^, ^70^, ^71^, ^72^ EMT has been shown to be involved in immune evasion of circulating tumour cells in multiple gastric cancer and breast cancer.^73^, ^74^ Ren et al. have reported that blocking of EMT signalling by inhibiting ZEB1 expression reverses chemoresistance in docetaxel‐resistant lung cancer cell lines.^75^ Moreover, EMT signalling can also promote tumour metastasis by regulating the expression of the oncogenic or tumour‐suppressive long non‐coding RNAs and proteins.^76^, ^77^ For instance, it has been reported that the EMT‐induced lncRNA NR2F1‐AS1 promoted gastric cancer metastasis via miR‐29a/VAMP7 axis.^78^ The interferon regulatory factor IRF6, which is repressed by EMT signalling, plays a tumour suppressive role in breast and gastric cancer.^79^ These findings implied that EMT signalling play critical roles in driving cancer metastasis in both direct and indirect manners.

## 3 MECHANISMS OF CELLULAR EMT SIGNALLING

The concept of EMT was first proposed by Green‐berg and Hay in 1982.^80^ After nearly 40 years of intensive research, the understanding of the occurrence of EMT has made remarkable progress.^56^ To date, hundreds of genes have been reported to be directly or indirectly involved in regulating the EMT signalling pathway. The molecular mechanisms by which these genes regulate cellular EMT signalling are diverse and cover various aspects, including pre‐transcriptional level, transcriptional level, post‐transcriptional level, translational level and post‐translational level.^81^, ^82^, ^83^, ^84^, ^85^, ^86^, ^87^ For example, at the pre‐transcription level, Dai et al. have reported that copy number gain of ZEB1 promoted EMT and bone metastasis in prostate cancer dependent on TGF‐beta signalling.^88^ Casalino et al. have summarized the role of DNA methylation in regulating cellular EMT by regulating protein‐coding and non‐coding genes.^89^ Recently, Shen et al. reported that EML4‐ALK G1202R mutation induces EMT by activation of STAT3/SNAI2 signalling in lung cancer.^90^ At the transcription and post‐transcription levels, increasing studies have confirmed that transcription factors as well as non‐coding RNAs involved into regulating the expression of EMT drivers play essential roles in cellular EMT in cancers. Our previous study reported that pseudogene lncRNA UBE2CP3 promotes gastric cancer EMT through sponging miR‐138, which repressed EMT signalling by targeting VIM.^91^ The m6A methyltransferase METTL3 promoted EMT in a m6A dependent manner in gastric cancer, lung cancer and nasopharyngeal carcinoma.^92^, ^93^, ^94^ At the translational and post‐translational levels, factors involved in the proteins stability of EMT‐related drivers, such as SNAI2 and ZEB1, functioned a role in cellular EMT. Zhang et al reported that CSN5 promoted renal cell carcinoma EMT by repressing ZEB1 degradation.^95^ Similarly, Zhou et al. found that USP51 overexpression promoted breast cancer EMT by deubiquitination and stabilization of ZEB1.^96^ Ouchida et al. reported that USP10 knockdown inhibited ovarian cancer EMT by regulating the protein stability of SNAI2.^97^

**FIGURE 3:**
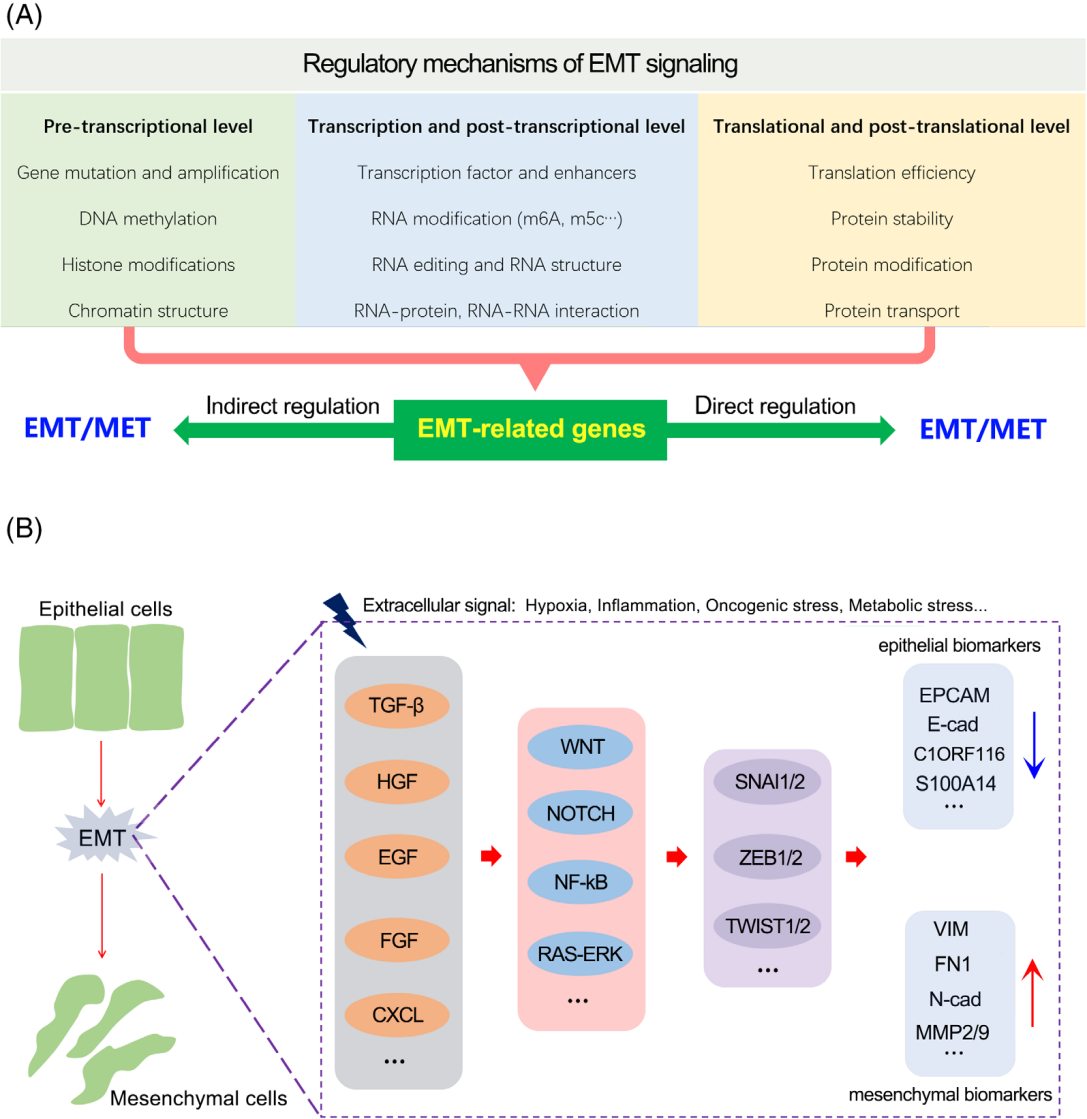
The regulatory mechanisms and signalling pathways of cellular EMT. (A) The way of gene regulating cellular EMT signalling pathway in cancers. (B) The signalling transduction pathways underlying cellular EMT.

The molecular mechanisms involved in EMT signalling are relatively well‐defined. When faced with stress conditions such as hypoxia, inflammation, oncogenic mutations and metabolic disorders, cells will activate the EMT signalling in response to external signals.^98^, ^99^, ^100^, ^101^ Activation of the EMT signalling requires a variety of humoral factors as extracellular messengers, including transforming growth factor (TGF‐β), fibroblast growth factor (FGF), hepatocyte growth factor (HGF), epidermal growth factor (EGF) and chemotaxis factor (CXC).^102^, ^103^ As the first messenger, once these extracellular signal molecules bind to receptors, they will transmit extracellular signals into the cell through Wnt, Notch, ERK, NFKB and other signalling pathways, and activate one or more EMT‐related transcription factors (EMT‐TFs), thereby regulating the expression level of EMT‐related target genes, and then splitting cell adhesion junctions to induce tumour cell invasion (Figure 3).^104^, ^105^, ^106^

Given that the EMT process is firmly induced by activation of signalling pathways, including but not limited to TGF‐β, Wnt/β‐Catenin, PI3K/AKT and Notch, targeting these signalling pathways could be promising strategies against cellular EMT signalling. Because of the extensive carcinogenic properties of these signalling pathways in cancers, many clinical drugs targeting these signalling pathways have been developed for anti‐tumour treatment. For example, lapatinib, an orally active drug for solid tumours through targeting HER2 and EGFR pathways, has been reported to inhibit the cellular EMT program in multiple cancers.^107^, ^108^, ^109^ Consistently, Clinical drugs targeting ALK, such as alectinib and lorlatinib, have been found to inhibit cellular EMT signalling in lung cancer.^110^ Gamma secretase inhibitors of Notch signalling has been confirmed to inhibit EMT in ovarian cancer.^111^ Therefore, it has become an increasingly promising anti‐tumour strategy by blocking EMT signalling pathway in cancer cells.^112^, ^113^

## 4 HETEROGENEITY AND PLASTICITY OF EMT

EMT is essentially an evolutionarily conserved program of cellular plasticity that controls the state of cells along the epithelial–mesenchymal axis, conferring EMT plasticity to epithelial cells.^41^, ^114^, ^115^ Epithelial–mesenchymal plasticity allows tumour cells to stay in different EMT transition states as needed.^116^ As summarized by Nam et al., EMT is a heterogeneous and dynamic disposition with intermediary or partial EMT meta‐states.^117^ When cells receive an EMT signal, they do not need to fully transition to the mesenchymal state, but can also maintain in diverse p‐EMT states.^45^, ^118^, ^119^ The p‐EMT state is also known as hybrid epithelial/mesenchymal state (hybrid E/M state or hybrid state) or intermediary state, and is located in the intermediate transition stage of epithelial and mesenchymal states.^55^, ^120^ And because of this, cells in hybrid EMT state exhibit both typical epithelial and mesenchymal characteristics. In other words, EMT is not a simple binary process, but absolutely contains a variety of continuous transition states, including epithelial state, mesenchymal state and different hybrid states.^61^, ^121^, ^122^

**FIGURE 4:**
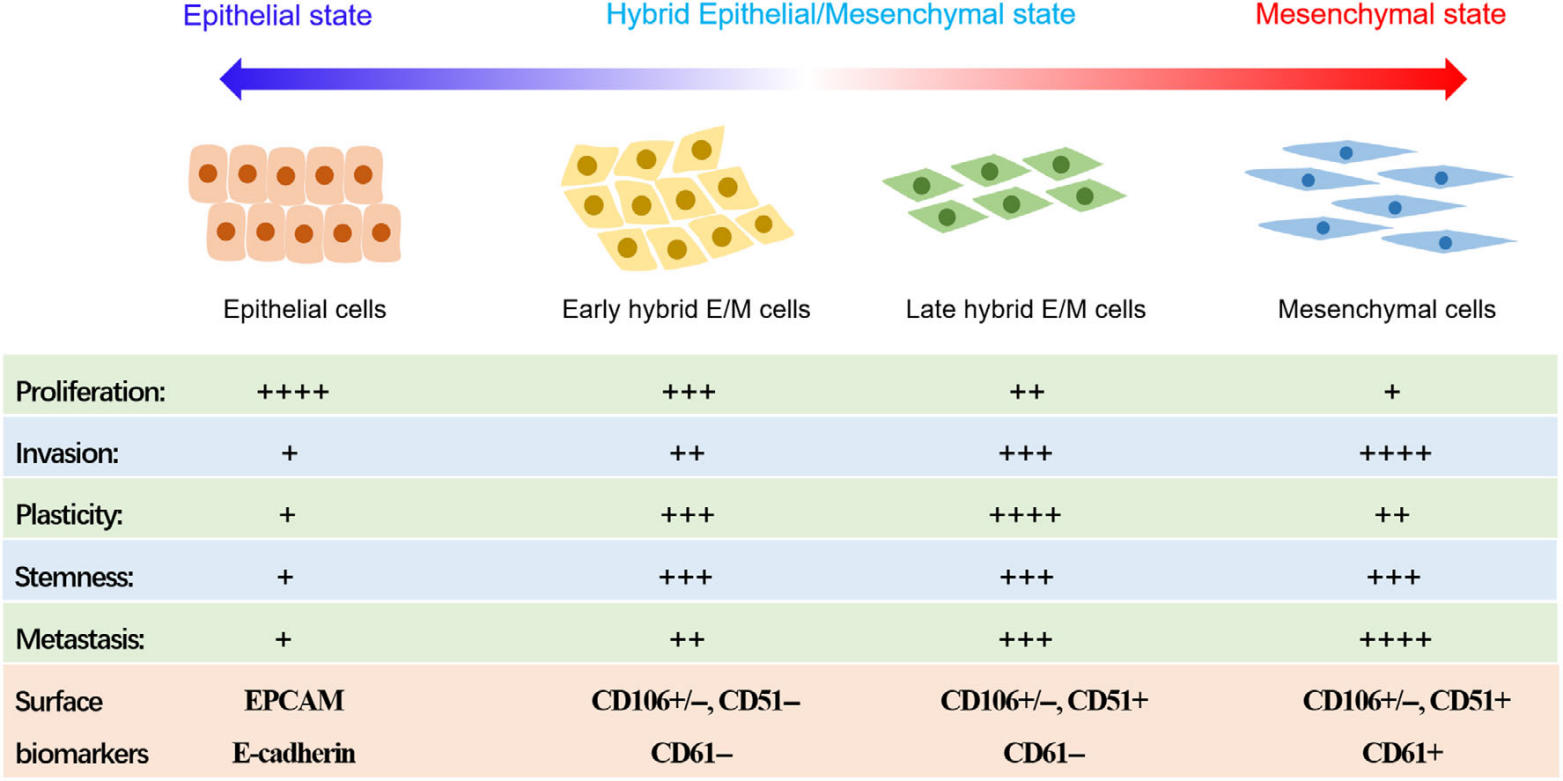
General characteristics of cells in different EMT transition states.

In 2018, Blanpain and his team systematically revealed the molecular characteristics, proliferation characteristics, invasion and metastasis characteristics, plasticity and stemness of cells under different EMT transition states (Figure 4).^32^ Weinberg and Blanpain and other teams have successively confirmed that compared with epithelial cells (high expression of E‐cad, Epcam, etc., with strong proliferation properties) and mesenchymal cells (high expression of VIM, etc., with strong invasive properties), cells with hybrid E/M phenotypes (capable of expressing both epithelial and mesenchymal markers) not only have strong tumour proliferation properties, but also have strong cell plasticity, stemness, invasion and metastasis properties.^55^, ^123^, ^124^

Emerging single‐cell sequencing studies have confirmed the dynamically configurable heterogeneity and plasticity of EMT.^31^, ^61^, ^62^, ^125^, ^126^ For example, Zhang et al. reported that p‐EMT signature genes were significantly enriched in malignant cells and predicted poor prognosis in skull base chordoma.^62^ Puram et al. have confirmed that cells with p‐EMT signature spatially localized to the leading edge of primary tumours, and were clinically associated with nodal metastasis, grade, and adverse pathologic features in head and neck cancer.^61^ Similarly, Simeonov et al. have confirmed that there are indeed different cell populations throughout the entire EMT process (from epithelial to hybrid and then to mesenchymal) in pancreatic and lung tumours by single‐cell RNA‐seq analysis.^31^ With the help of pedigree tracking using the macsGESTALT method and single‐cell RNA‐seq analysis, they found that pancreatic cancer cells run through different states of the whole continuous EMT, including epithelial state, different hybrid states and mesenchymal state, suggesting that cellular EMT is actually a continuous process. Besides, they further revealed that the gene signatures of late‐hybrid EMT status predicted poor prognosis in human pancreatic and lung cancer.^31^

Although most spontaneous tumours originate from a single cell, the mechanism of maintaining the clonal evolution of normal progenitor cells does not seem to play a full role in tumours.^127^ Most human tumours show incredible heterogeneity in numerous morphological and physiological characteristics, such as invasion, proliferation and angiogenesis potential, ultimately leading to the differences of clinical manifestations, therapy resistance and prognosis in patients.^128^ Molecular typing studies have confirmed that there are significant histological, transcriptomic and genomic differences among certain cancer patients, which are also known as inter‐patient heterogeneity.^129^, ^130^ In addition, recent single‐cell RNA sequencing (scRNA‐seq) studies have further revealed that there was also a high degree of heterogeneity in the cell population in an individual tumour, called intra‐patient heterogeneity.^131^

**FIGURE 5:**
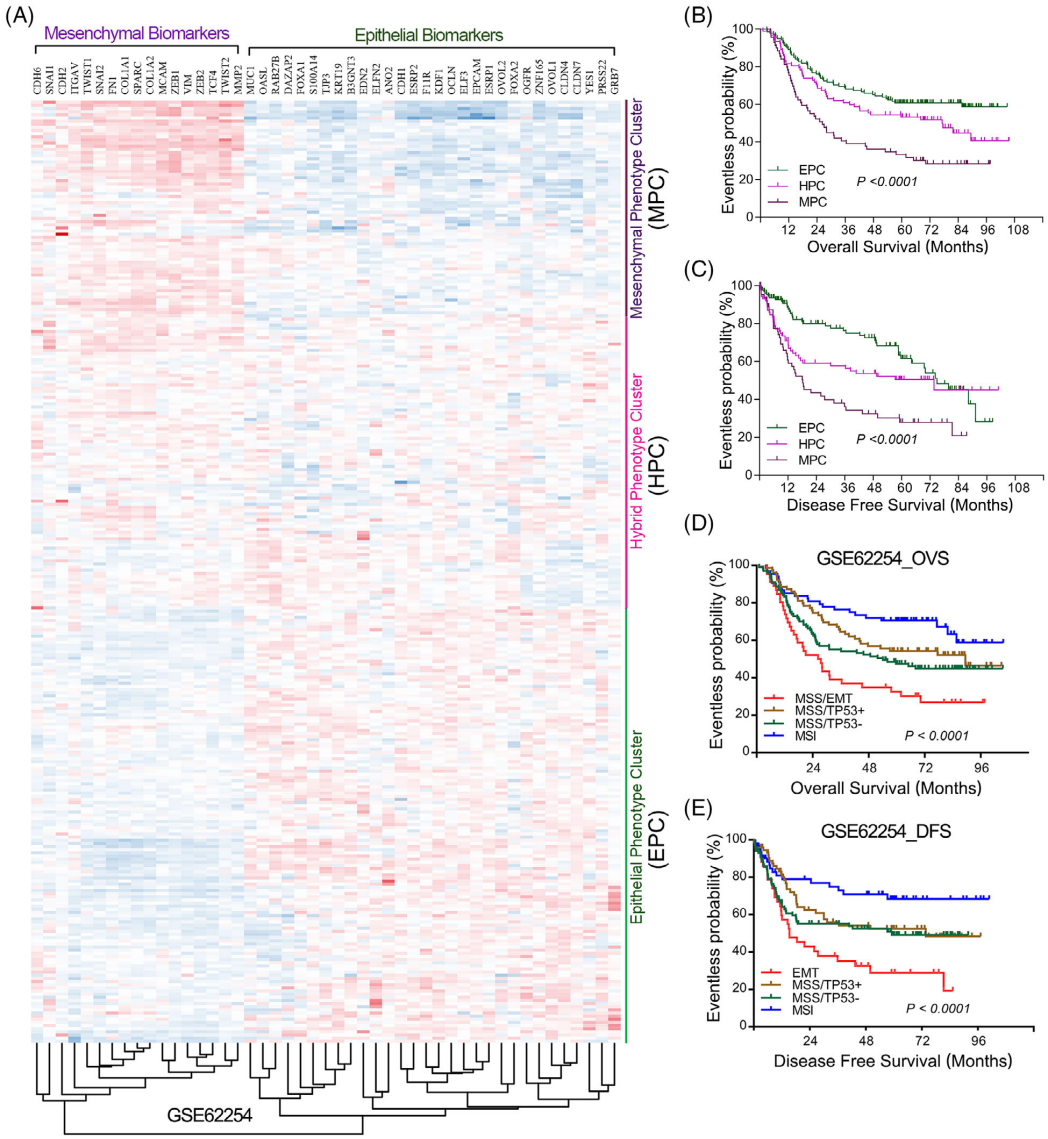
Clustering gastric cancer patients based on the expression patterns of EMT‐related genes in GSE62254 cohort. (A) Based on the expression patterns of EMT‐related genes, we performed EMT‐related molecular typing in 300 gastric cancer patients. According to the degree of EMT progression, GC can be further subdivided into epithelial phenotype cluster (EPC, *n* = 139), hybrid phenotype cluster (HPC, *n* = 93) and mesenchymal phenotype cluster (MPC, *n* = 69). (B, C) The overall survival (OVS) and disease‐free survival (DFS) analysis of gastric cancer patients in EPC, MPC and HPC. (D, E) The overall survival (OVS) and disease‐free survival (DFS) analysis of gastric cancer patients in MSS/TP5−, MSS/TP53+, MSI and MSS/EMT subtypes.

The degree of epithelial–mesenchymal plasticity of tumour cells is continuous and diverse. To a substantial extent, the inter‐tumour and intra‐tumour heterogeneity might be attributed to the proportion of cancer cells staying in various EMT transformation states within and between tumours. We chose to test this hypothesis in gastric cancer, a highly heterogeneous tumour.^132^ The inter‐tumour heterogeneity of gastric cancer was investigated by molecular typing gastric cancer patients based on the expression patterns of EMT‐related genes. The EMT‐related molecular typing studies were performed in the GSE62254 gastric cancer cohort.^133^ The classic epithelial marker genes and mesenchymal marker genes were selected to cluster 300 gastric cancer patients in GSE62254 cohort. Molecular typing results showed that gastric cancer can be further subdivided into three subtypes according to the degree of EMT progression (Figure 5A), including epithelial phenotype cluster (EPC, *n* = 138), hybrid phenotype cluster (HPC, *n* = 93) and mesenchymal phenotype cluster (MPC, *n* = 69). Survival analysis additionally revealed that gastric cancer patients in MPC cluster possessed poorest overall survival and disease‐free survival, while gastric cancer patients in EPC cluster possessed best overall survival and disease‐free survival in GSE62254 cohort (Figure 5B,C). Similarly, Cristescu et al. have reported that GSE62254 cohort could be further divided into four subtypes, including MSS/TP5−, MSS/TP53+, MSI and MSS/EMT subtypes.^133^ Consistently, our survival analysis fitted extremely nicely with their conclusion that gastric cancer patients in EMT subtype possessed the poorest prognosis (Figure 5D,E). These findings firmly suggested that the inter‐tumour heterogeneity of gastric cancer was attributed at least in part to the heterogeneity of EMT progression in patients.

## 5 THE REGULATORY MECHANISM OF DIFFERENT EMT TRANSITION STATE

Most previous studies on EMT/MET signalling focused on how cells transition from an epithelial (mesenchymal) to a mesenchymal (epithelial) state. However, relatively little is known about exactly how cells transition from an epithelial/mesenchymal state to an intermediate state. For example, Tian et al. found that double negative feedback loops play essential roles in regulating EMT transition states through mathematical model research.^134^ Subsequently, Mohit Kumar Jolly's team successively confirmed that OVOL, SLUG and GRHL2 and other double‐negative feedback loops are involved in the fine regulation of EMT transition state. Recently, Mombach's team found that the fine regulation of EMT‐TFs expression mediated by double‐negative feedback loops is necessary for EMT phenotype stabilization.^135^

**FIGURE 6:**
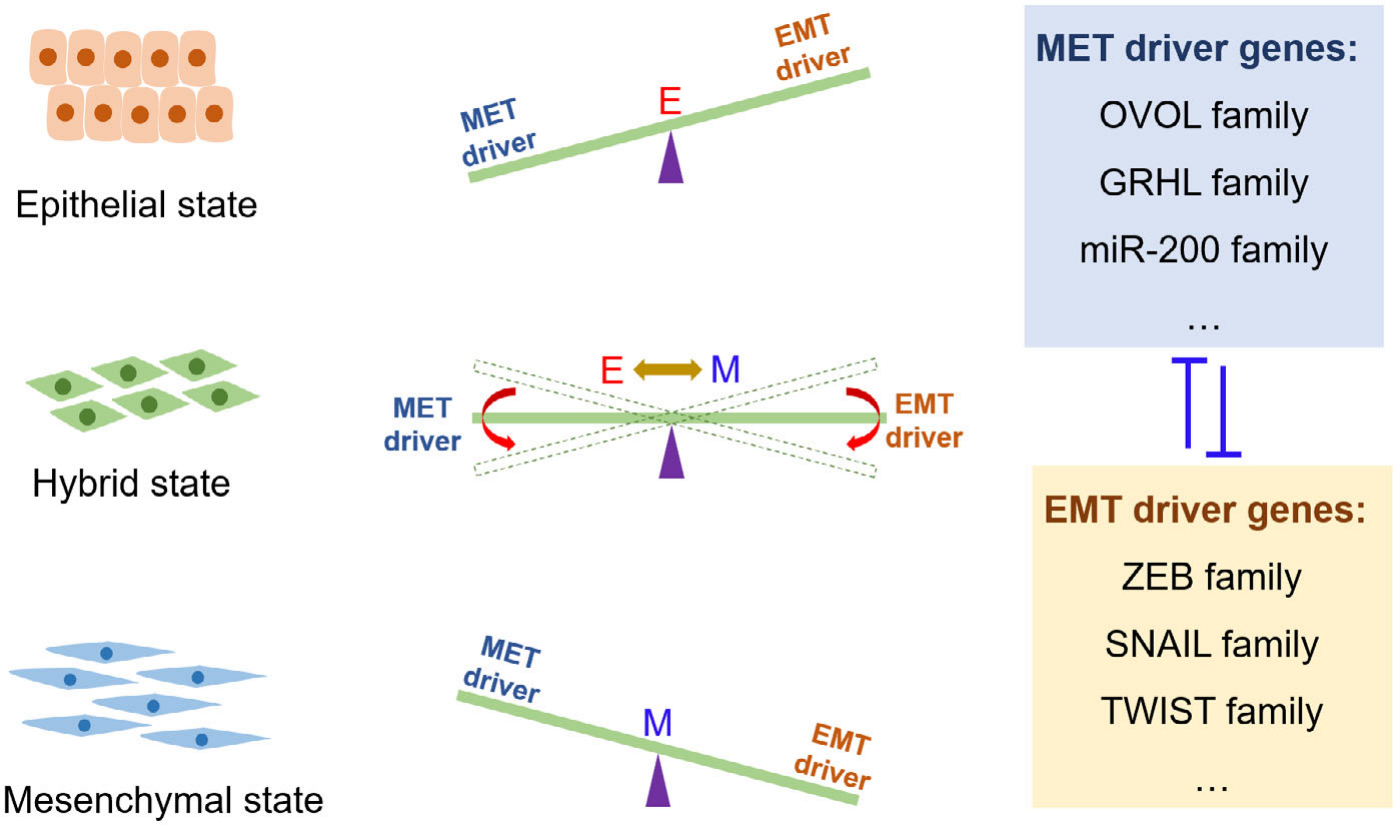
The seesaw model between EMT driver genes and MET driver genes was proposed to explain the underlying molecular mechanism of the generation of different EMT transition states.

To date, multiple double‐negative feedback loops have been identified in the cellular EMT signalling pathway, including ZEB1/OVOL2, GRHL2/ZEB1, ZEB1/miR‐200, miR‐34/SNAILs and miR‐203/SNAILs.^136^, ^137^, ^138^, ^139^, ^140^ These double‐negative feedback loops play critical roles in finely regulating the expression of the master regulators of EMT signalling, such as ZEBs and SNAIs. These double‐negative feedback loops in the EMT signalling pathway can be seen as a seesaw. The two ends of the seesaw are EMT driver genes and MET driver genes. The EMT driver and the MET driver “wrenches” with each other through a double negative feedback loop. According to the result of the wrestling, the cells may end up in the following three states. If the EMT (MET) drivers are completely dominant, the cells will remain in the mesenchymal state (epithelial state). Conversely, if neither the EMT drivers nor the MET drivers have achieved dominance, the cells will stay in the hybrid E/M states (Figure 6).

Theoretically, a single double‐negative feedback loop is sufficient to keep the epithelial/mesenchymal cells stably in epithelial/mesenchymal, whereas the p‐EMT state appears to be transient and unstable. Once the expression of the EMT/MET driver is up‐regulated, the expression of the MET/EMT driver will be down‐regulated under the influence of the feedback loop, which further promotes the up‐regulation of EMT/MET driver, ultimately achieving a stable mesenchymal/epithelial state. However, this is not entirely the case, as p‐EMT cells can actually be stable in tumours. For instance, MCF10A has been reported to be a breast cancer cell line in p‐EMT state because it has moderate levels of EMT‐related gene expression.^137^ This suggests that the double negative feedback loops in the EMT signalling pathway are not isolated, but crosstalk each other and are finely regulated. For example, the OVOL2‐ZEB1, and ZEB1‐miR‐200 loops have crosstalk due to overlap. Hong et al. have found that the OVOL2‐ZEB1, ZEB1‐miR‐200 and SNAI1‐miR‐34a loops were all contribute to the existence and robustness of the p‐EMT states in breast cancer, and the strength of the Ovol2‐Zeb1 loop is more critical.^137^ In summary, the combined action of multiple double‐negative feedback loops may be required to keep cells in a relatively stable p‐EMT state.

**FIGURE 7:**
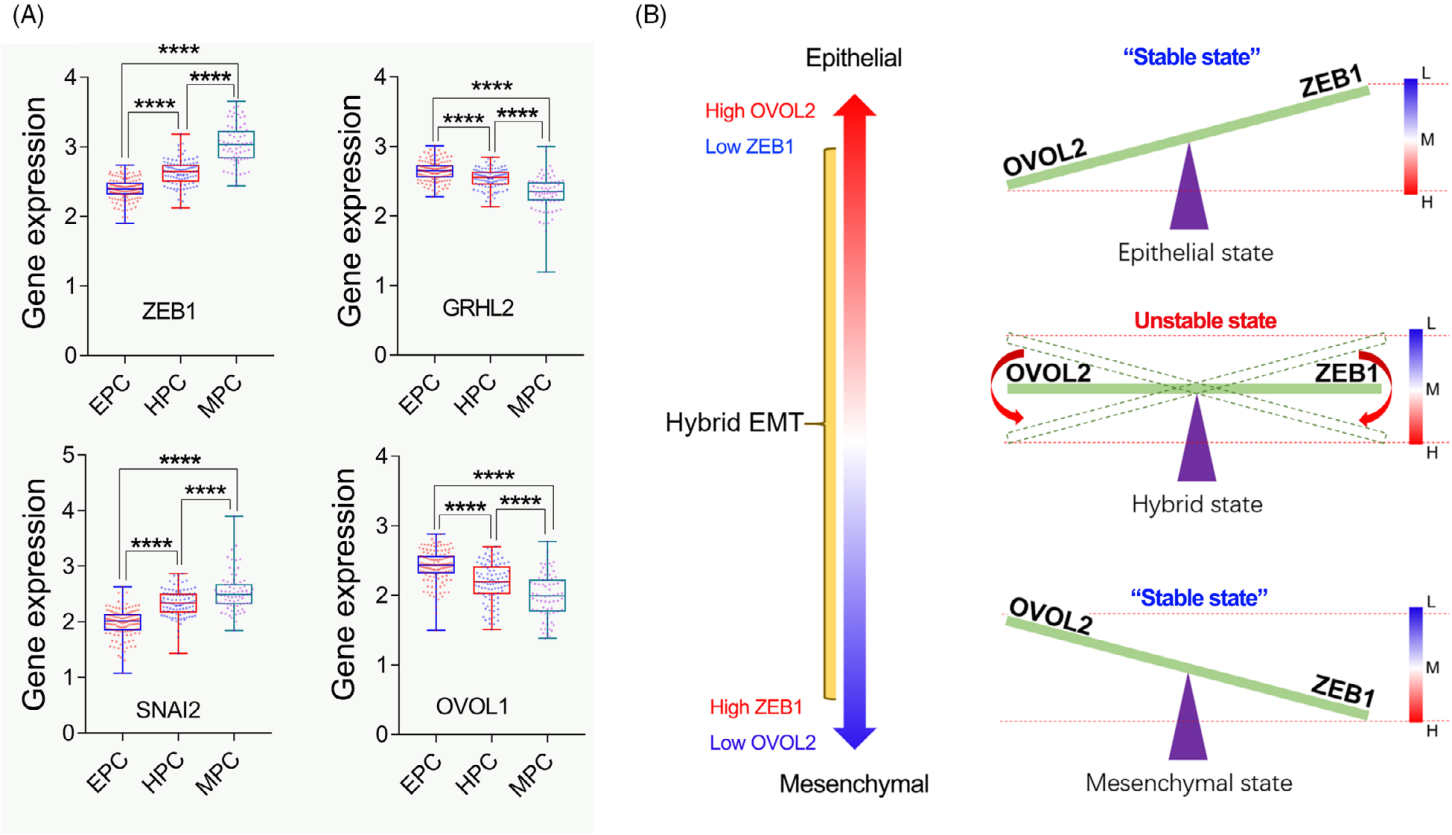
The seesaw model was proposed to explain the EMT heterogeneity in gastric cancer. (A) The expression changes of EMT driver genes (ZEB1 and SNAI2) and MET driver genes (GRHL2 and OVOL1) in different subtypes of GC patients. (B) According to our seesaw model, cells highly expressing ZEB1/SNAI2 were in mesenchymal state, cells highly expressing GRHL2/OVOL1 were in epithelial state, cell moderately expressed ZEB1, SNAI2, GRHL2 or OVOL1 were in hybrid state.

To validate our proposed seesaw model, we compared the expression levels of EMT drivers (ZEB1 and SNAI2) and MET drivers (GRHL2 and OVOL1) in the different EMT subtypes of GSE62254 cohort.^133^ The results showed that the expression levels of EMT driver genes were significantly increased, while the expression levels of MET driver genes were significantly decreased in the sequential EMT transition state (Figure 7A). The tumour cells in the epithelial state possessed elevated expression levels of GRHL2 and OVOL1, low expression levels of ZEB1 and SNAI2. Conversely, tumour cells in the mesenchymal state possessed low expression levels of GRHL2 and OVOL1, high expression levels of ZEB1 and SNAI2. Tumour cells in a hybrid E/M state possessed moderated expression levels of GRHL2, OVOL1, ZEB1 and SNAI2 (Figure 7B). The results implied that the proposed seesaw model can reasonably explain the generation of different EMT transition states in cells.

## 6 CURRENT STATUS AND PERSPECTIVE OF EMT

Although the heterogeneity of EMT has been divided into different transition states, including epithelial state, p‐EMT/hybrid or intermediate state (early intermediate state, intermediate state, late intermediate state) and mesenchymal state. Admittedly, the intermediate state of EMT is actually continuous, with great heterogeneity and plasticity. In addition, recent single‐cell analysis has confirmed that even the epithelial or mesenchymal states at either extremes of EMT have obvious heterogeneity. For example, several independent single‐cell analyses have shown that there is still a high degree of marked heterogeneity in the epithelial state cells of gastric cancer.^141^, ^142^, ^143^ These studies implied that the heterogeneity and plasticity of EMT is far more complex than we thought. The high heterogeneity of EMT may be partially attributed to the multi‐level regulation of different signalling pathways and their crosstalking.

The redundant and non‐redundant functions of EMT‐TFs and their clinical significances remain poorly understood. EMT signalling is directly regulated by various EMT‐TFs.^144^ The dysregulation of EMT‐TFs results in aberrant activation of EMT signalling in cancer metastasis.^145^ To date, multiple families of transcription factor have been identified that can directly regulate EMT signalling in cells, including ZEBs, SNAILs, OVOLs, TWISTs, and GRHLs.^146^, ^147^, ^148^ Interestingly, these transcription factor families all contain multiple members, such as ZEB1/2, SNAI1/2/3, OVOL1/2/3 and GRHL1/2/3. There must be redundant and non‐redundant functions among these homologous members of the EMT‐TF family.^149^, ^150^ However, how this functional redundancy among members within the family affects the corresponding double negative feedback loop remains unclear. For example, it has been reported that Snail transcription factors possessed partially functional redundancy in haematopoietic cell development and tumour metastasis.^151^, ^152^ Interestingly, SNAI1 and SNAI2 directly inhibited each other by binding both their own and each other's promoter in chondrogenesis and cancer metastasis, suggested that there is a double‐negative feedback loop between SNAI1 and SNAI2.^153^, ^154^, ^155^ Since SNAI2 and SNAI1 were both the target gene of miR‐34a,^156^ it is necessary to evaluate the regulatory relationship between SNAI2/SNAI1 and SNAI1/miR‐34 loops. One possible explanation is that the mutual inhibition of SNAI1 and SNAI2 to fine control the overall expression levels of the SNAIL family.

According to the seesaw model, the fine regulation of EMT‐TF expression is required to maintain a stable EMT transition state in cells. On the one hand, the expression of EMT‐TF is regulated by corresponding double‐negative feedback loops. Although multiple double‐negative feedback loops in the EMT signalling have been identified in cellular EMT signalling pathways, an intriguing phenomenon is that EMT‐TFs are directly involved in all these loops. On the other hand, the expression of EMT‐TFs is modulated by functional redundancy among members of the EMT‐TF family. These EMT‐TFs families have multiple functionally redundant members. However, it is unclear how functional redundancy among members of the EMT‐TF family and these double‐negative feedback loops synergistically regulate cellular EMT transition states. Furthermore, due to the tissue specificity of gene expression, it is inevitable that a certain loop cannot play a key role between tissues. Therefore, there may be different dominant circuits in the EMT signalling pathways of different cells or tissues, which need to be further explored.

In addition to promoting tumour metastasis, EMT also plays critical roles in cancer cell stemness, drug resistance and immune escape.^113^, ^157^ Theoretically, blocking EMT signalling is a promising anti‐tumour strategy that “kills many birds with one stone.” The current clinical drugs are typically designed to block a single EMT signal pathway, such as lapatinib targeting EGFR and alectinib targeting ALK. However, due to the frequent cross‐talking among the different EMT signalling pathways, it is difficult to effectively inhibit cancer metastasis by targeting a single EMT signalling pathway. In addition, for a certain tissue, multiple EMT pathways may exist simultaneously. However, when the major EMT signalling pathway is blocked, the “secondary EMT pathway” at that time becomes the “major EMT pathway,” thereby resulting in resistance.^158^, ^159^, ^160^ Therefore, developing drugs that can target multiple EMT signalling pathways is a feasible research direction in the future.^161^, ^162^ Notably, although there are multiple pathways that can induce cellular EMT, one common feature of these signalling pathways is that they need to activate EMT‐related transcription factors, such as ZEBs, SNAIs or Twists.^105^ Targeted inhibition of EMT‐TF function may be one of the best ways to block cellular EMT in cancer cell. Interestingly, Watanabe et al. have reported a significant transcription repression on those EMT‐TFs (including Zeb1/2, Snai/2, Vim and Twist1) by MET inducer Ovol2 in mice.^163^ Perhaps developing drugs to reactivate the expression of OVOL2 and thus induce MET in tumour cells would be an excellent option for anti‐metastasis.

## AUTHOR CONTRIBUTIONS

Dandan Li: conceptualization, methodology, writing—original draft, investigation. Lingyun Xia: conceptualization, methodology, software, writing—original draft, investigation. Pan Huang: investigation, resources, supervision. Zidi Wang: investigation, resources, supervision. Qiwei Guo: methodology, investigation. Congcong Huang: resources, software. Weidong Leng: conceptualization, writing—review and editing, resources, supervision, project administration and funding acquisition. Shanshan Qin: conceptualization, methodology, writing—original draft, investigation, writing—review and editing, resources, supervision, project administration and funding acquisition.

## ACKNOWLEDGEMENTS

We thank the relevant Gene Expression Omnibus repository for providing the data for RNA‐seq analysis.

## CONFLICT OF INTEREST STATEMENT

The authors declare that they have no competing interests.

## Data Availability

Data supporting the findings of this study are available from the corresponding author upon reasonable request.
